# Influence of Ionic Surfactants on Microfiltration of Enveloped and Nonenveloped Viruses

**DOI:** 10.1002/wer.70171

**Published:** 2025-09-02

**Authors:** Makayla Loey, Damien Redder, Emily L. Marron, Jennifer Weidhaas

**Affiliations:** ^1^ Department of Civil and Environmental Engineering University of Utah Salt Lake City Utah USA; ^2^ Water Resources Research Center University of Hawaii at Manoa Honolulu Hawaii USA

**Keywords:** enveloped and nonenveloped viruses, log reduction value, microfiltration, surfactants, wastewater, water treatment

## Abstract

Viruses in wastewater effluent pose significant public health risks, and achieving high log reduction values is critical for wastewater reuse applications. The influence of ionic surfactants sodium dodecyl sulfate (SDS) and benzyldimethyldodecylammonium chloride (BAC) in wastewater on virus removal and infectivity after microfiltration (200 nm cutoff) was investigated. Microfiltration log rejection values (LRVs) for infective human adenovirus (ADV, nonenveloped, 90‐nm diameter) and mouse hepatitis virus (MHV, enveloped, 80‐ to 120‐nm diameter) increased when surfactants were present in wastewater from 4.3 to 6.8–7.1 and from 4.8 to 4.9–5.1 LRV for ADV and MHV, respectively. Lower LRVs were found when using quantitative polymerase chain reaction (qPCR) than by TCID_50_/mL infectives for both viruses, specifically from 1.4 to 3.4 for ADV and MHV, respectively. SEM‐EDS confirmed aggregated virus and surfactant sorption on the membrane. This work advanced understanding of microfiltration virus log reduction influenced by surfactants and is applicable to wastewater reuse.

## Summary

- Ionic surfactants, sodium dodecyl sulfate (SDS), and benzyldimethyldodecylammonium chloride (BAC) increase rejection of viruses during microfiltration.
- Nonenveloped adenovirus rejection by microfiltration improved from 4.3 to 6.8–7.1 log when surfactants are present
- Enveloped and nonenveloped virus infectivity decreased by 3 orders of magnitude during microfiltration in the presence of 1 mg/L surfactants.
- Virus aggregation and sorption on microfilter were observed by SEM‐EDS.

## Graphical abstract

Low concentrations of ionic surfactants, sodium dodecyl sulfate (SDS), and benzyldimethyldodecylammonium chloride (BAC), in water systems, increase rejection of viruses during microfiltration by 2–3 log and decrease virus infectivity by 3 log.

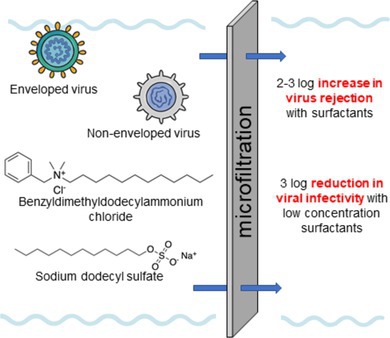

## 1 Introduction

Viruses can persist in treated wastewater effluent, with an average of 10^4^ virus genome copies/L (Corpuz et al. 2020). These viruses represent a significant risk to human health (Gall et al. 2015) as treated wastewater might be reused for irrigation, released into surface water, or even further treated for reuse as drinking water (Sano et al. 2016). To minimize public health risks from viruses in water reuse scenarios, treatment processes must be highly effective at removing or inactivating viruses (Al‐Hazmi et al. 2022). Additionally, surface‐active agents, such as surfactants from personal care and cleaning products, have been consistently detected in domestic wastewater influents at concentrations ranging from 0.04 μg/L to 10 mg/L (Clara et al. 2007; Bautista‐Toledo et al. 2014; Bergé et al. 2018; Mongelli et al. 2025). These ionic surfactants, which are either anionic, cationic, or zwitterionic based on the charge of their hydrophilic head, can interact with viral surface membrane proteins or adsorb to filtration membranes used in water treatment processes (Cavanaugh and Weidhaas 2023b).

Technologies for removing viruses and other microorganisms from wastewater include disinfection and biochemical processes and membrane filtration, each offering several advantages and disadvantages (Bodzek et al. 2019). Biochemical processes, such as activated sludge and anammox‐based systems, primarily target the removal of organic matter and nutrients, with some limited ability to remove viruses (Al‐Hazmi et al. 2022). However, the efficacy of viral removal through the biochemical processes is low; for instance, biochemical processes have been reported to only remove 1‐log adenovirus ds DNA and less than 1‐log of most RNA viruses (Al‐Hazmi et al. 2022). To achieve higher viral removal efficiencies, biochemical processes are often paired with complementary treatments in wastewater treatment plants. Disinfection methods such as UV radiation, chlorination, and ozone treatment have a higher viral removal or inactivation efficacy (Al‐Hazmi et al. 2022). However, disinfection processes could create disinfection by‐products (DBPs) due to incomplete chemical reactions with the organic or inorganic precursors (Bergé et al. 2018; Carrillo et al. 2018). With the growing concern over viral infectivity following disinfection, adopting alternative or supplementary virus removal methods is crucial for effective wastewater treatment (Al‐Hazmi et al. 2022).

Membrane filtration is an effective approach to improve virus removal from wastewater as part of a larger treatment system (Bodzek et al. 2019) or in combination with biological treatment in a membrane bioreactor (Zheng and Liu 2007). These filters rely on various membrane rejection mechanisms, including size exclusion, steric hindrance, electro‐steric attraction, and internal impaction to effectively reject viruses and contaminants in water (Cui et al. 2010; Cavanaugh and Weidhaas 2023a, 2023b; Mahmoud and Mostafa 2023). Membrane filter categories include microfiltration, ultrafiltration, nanofiltration, and reverse osmosis. Based on the membrane pore size and surface characteristics, ultrafiltration (0.1‐μm or 100‐nm cutoff size) and nanofiltration (0.001‐μm or 1‐nm cutoff size) membranes are generally suitable for removing unaggregated viruses from water (Bodzek et al. 2019). Utilities with membrane bioreactors as part of their water reuse treatment trains typically utilize microfiltration (MF) membranes, which typically have pore sizes larger than average virus diameters (Jefferson et al. 2000). Microfiltration membranes used in membrane bioreactors have reported virus log removal factors ranging from 1 to 6 (Zheng and Liu 2007; O'Brien and Xagoraraki 2020). Several reports have also noted the ability of membrane bioreactors to remove surfactants from wastewater (González et al. 2007; Gori et al. 2010). Virus rejection in microfiltration membranes has been associated with viruses exhibiting colloidal activity (Zhdanov 2021), virus aggregation increasing the virus apparent diameter, membrane surface‐virus electrostatic rejections (Sinclair et al. 2018; Yasui et al. 2024), and foulants on membrane surface decreasing the membrane pore size (Sinclair et al. 2018; Yasui et al. 2024).

The Derjaguin, Landau, Verwey, and Overbeek (DLVO) theory offers valuable insights for predicting the interaction between viruses and surfaces during membrane filtration, based on the balance between attractive (van der Waals) and repulsive (electric double‐layer) forces between virus particles and membrane surface (Agmo Hernández 2023). According to DLVO theory, the strength of the attraction and repulsion forces significantly influences the efficiency of viral rejection, as they determine whether particles adhere to or are repelled by the membrane surface and pores. In this context, we propose that the presence of ionic surfactants in wastewater would alter the rejection efficiency of viruses, compared to solutions without surfactants. Ionic surfactants, due to their charged properties, might interact with both the virus particles and membrane surface, potentially modifying the balance of forces and impacting virus retention. This interaction might either enhance or inhibit virus rejection, depending on the charge, concentration, and type of surfactant present. Further, virus rejection might also be influenced by the pH of the solution and isoelectric point (IEP or the pH at which the net charge of the virion is zero) of the virus as modified by interactions with the ionic surfactant.

The overall objective of this research was to investigate the interactions between surfactants and viruses that might alter virus surface electrostatic properties and further influence membrane filtration efficacy. For most nonenveloped viruses, the isoelectric point is between 2 and 7 depending on ionic strength and pH of the wastewater (Heffron and Mayer 2021). In the case of the enveloped virus, the surface charge of the capsid is equally important, which controls the interactions with the lipid bilayers (Duran‐Meza et al. 2021). It was hypothesized that surfactants would affect the efficacy of virus removal during microfiltration by altering the charges on both the virus and membrane surface. This study focused on virus removal efficacy and used both enveloped mouse hepatitis virus (MHV‐A059, hereafter MHV) and nonenveloped human adenovirus (ADV‐05, hereafter ADV). The respective size and isoelectric point (IEP) of these viruses are shown in Table 1.

**TABLE 1:** Characteristics of viruses included in the study.^a^

Name	Mouse hepatitis virus (MHV‐A59)	Human adenovirus (ADV‐05)
Virus type	Enveloped ss RNA	Nonenveloped ds DNA
Diameter^b^	80–120	90
Empirical IEP	4.2	3.8

^a^ Bárcena et al. (2009) and Heffron and Mayer (2021).

^b^ Takuissu et al. (2024).

**FIGURE 1:**
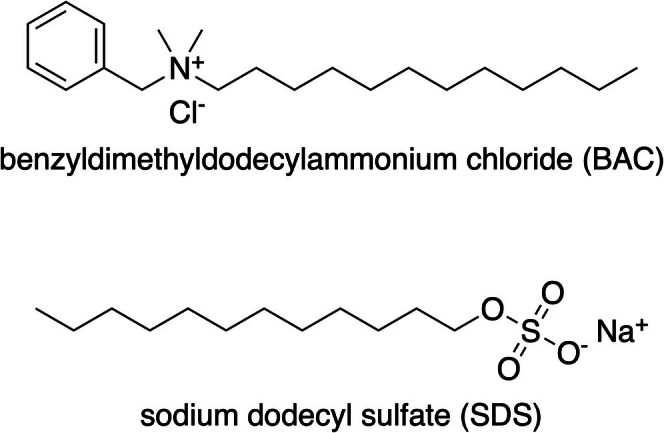
Chemical structures of benzyldimethyldodecylammonium chloride (BAC) and sodium dodecyl sulfate (SDS).

This study investigated the impact of representative ionic surfactants on virus rejection in a microfiltration system, specifically the positively charged surfactant benzyldimethyldodecylammonium chloride (BAC) and negatively charged surfactant sodium dodecyl sulfate (SDS) (see Figure 1). These surfactants were selected to explore how charged interactions between surfactants, virus capsid or envelope proteins, and membrane surfaces impacted virus rejection. Concentrations of BAC have not been reported in the literature to date, but related surfactants with longer hydrocarbon chains have been detected in wastewater influent up to 170 ug/L for benzyldimethyldodecylammoniumchlorid (BAC‐C_12_) to 110 ug/L for denzyldimethyltetradecylammonium chloride (BAC‐C_14_) (Clara et al. 2007). SDS has long been known to be present in wastewater influent with reports as early as the 1990s in concentrations of 180 ug/L (Fendinger et al. 1992).

## 2 Methodology

### 2.1 Wastewater Collection and Handling Methods

Wastewater effluent samples used for microfiltration studies were collected from the Central Valley Wastewater Reclamation Facility (CVWRF) in Salt Lake City, Utah. A total of 7 L of wastewater was collected from the secondary effluent in sterile 1‐L polypropylene (Wong et al. 2012) bottles and stored at 4°C until use (within 7–10 days). All wastewater was filtered through a sterile 0.45‐μm nitrocellulose membrane filter paper (Merck KGaA, Darmstadt, Germany), and the pH was adjusted to 7 using hydrochloric acid and sodium hydroxide during microfiltration experiments. The abundance of MHV and ADV in the wastewater, as well as the concentrations of SDS and BAC in the secondary wastewater effluent, was quantified before spiking with additional surfactant and/or viruses. All samples were handled in a biosafety level 2 (BSL‐2) laboratory equipped with a biosafety cabinet per institutional biosafety committee approved protocols.

### 2.2 Cell Culture and Virus Propagation

Cell lines were purchased from the American Types Culture Collection (ATCC, Manassas, Virginia, United States). The cell lines of HEK‐293 (human kidney epithelial cells) and L929 (mouse fibroblast connective tissue cells) were cultured for propagating ADV and MHV, respectively. The original viruses were purchased from ATCC (Manassas, Virginia, United States). Both cell lines were cultivated using 75‐cm^2^ U‐shaped vented flasks (Sigma‐Aldrich, St. Louis, Missouri, United States), which contained 13.5 mL of Eagle's Minimum Essential Medium (EMEM) (ATCC) and 1.5 mL of either fetal bovine serum for ADV or horse serum for MHV (MP Biomedical Inc., Santa Ana, California, United States). Cell lines were incubated at 37°C in a 5.5% CO_2_ atmosphere (VWR, Radnor, Pennsylvania, United States). Cell subculturing was handled by trypsinization when cells reached 90% confluence, as observed using invented microscopy (MU 900 AmScope, Irvine, California, United States). A total of 3 mL of 0.25X trypsin [ATCC] solution was used to dissociate cells from the flask bottom for 7 mintes. Then, 7 mL of cell medium was used to end the trypsinization. Cells were collected and centrifuged at 1500 rpm for 5 min. At the end of the centrifugation, the cell pellets will be resuspended in 5 mL of cell medium and distributed into five new flasks.

Cells were stored in liquid nitrogen after preparation according to the methods published previously (Shuipys and Montazeri 2022), with the following exceptions. Prior to freezing, the cell pellets were resuspended in a Recovery Cell Culture Freezing Medium (Fisher Scientific, Waltham, Massachusetts, United States). A 2‐CHIP cell counting plate (Bulldog‐Bio, Portsmouth, New Hampshire, United States) ensured the cell counts reached 10^6^ cells in 1 mL before storage. Mr. Frosty Freezing Container (Thermo‐Fisher Scientific, Waltham, Massachusetts, United States) filled with isopropanol (Sigma‐Aldrich) was used to facilitate the cell cooling process in a −20°C laboratory standard freezer (VWR) before transferring the container to a −80°C freezer (Thermo Scientific, Waltham, Massachusetts, United States) for an additional 24 h to achieve an ideal rate of cooling. Finally, cells in the Nalgene cryogenic vial (Sigma‐Aldrich) were transferred to a liquid nitrogen tank for storage.

To propagate viruses, 3 mL of existing MHV or ADV in the virus medium was added to a 75‐cm^2^ flask containing the appropriate cell lines. Then, the cells and viruses were incubated at 37°C in 5.5% CO_2_ for either 1 h for MHV or 2 h for ADV, to assist cell infection with viruses. Then, 22 mL of a 9:1 volumetric ratio of NCTC‐135 virus growth medium (Sigma‐Aldrich) and horse serum for MHV and 19.6 mL of EMEM with 0.4 mL of FBS for ADV were added to the flask to assist virus propagation for 18–24 h (MHV) or 24–48 h (ADV). Upon completion of virus harvesting, all solutions were transferred to a 50‐mL centrifuge tube (Corning, Corning, New York, United States) and frozen at −80°C for 20–30 min, followed by thawing to optimize intracellular virus particle release. Subsequently, centrifugation at 3000 rpm for 10 min (5910R centrifuge Eppendorf, Hamburg, Germany) was employed to precipitate all cell debris, and the supernatant virus solution was collected for further virus purification.

### 2.3 Virus Purification From Cell Culture

Before microfiltration, the virus stock solution harvested from cell culture was first concentrated using a 20% sucrose solution (Sigma Aldrich) in deionized (DI) water. In each ultracentrifugation tube (Seton Scientific, Petaluma, California, United States), 8 mL of viral stock solution was first placed into the tube, followed by injecting 1 mL of 20% sucrose solution into the bottom of each tube using a long‐tip pipette. The 8‐mL virus sample was centrifuged in a swinging bucket rotor ultracentrifuge (L8‐70 M, Beckman, Brea, California, United States) at 20,000 rpm (70,000×*g*) for 2 h at 4°C. Upon completion, the supernatant was removed from each ultracentrifugation tube. The tube was then dried using sterile Q‐tips (Medline, Northfield, Illinois, United States). A 100 μL of 1X phosphate buffer saline (PBS) solution (i.e., 137 mM NaCl, 10 mM phosphate, 2.7 mM KCl; pH 7.4) was added to each tube to assist in precipitating the concentrated virus pellet overnight. The next day, the purified viruses were resuspended and transferred into 2‐mL PCR tubes, which were ready for microfiltration. There is a potential that the viruses may have aggregated during this sucrose concentration method, but this was not evaluated during this study.

### 2.4 Membrane Microfiltration System

The membrane filtration system was a cross‐flow setup (Sterlitech, Auburn, Washington, United States) that has been previously described (Cavanaugh and Weidhaas 2023a, 2023b). Herein, the studies were conducted with a polyethersulfone membrane (PES 020, Sterlitech) with an average pore size of 0.2 um. The membrane pore size was documented by the vendor and confirmed using scanning electron microscopy (SEM) (Zeiss Gemini 300, Oberkochen, Germany) herein. Before the filtration experiment, the membrane was hydrated using 200 mL of DI water. To maintain the retentate temperature between 15°C and 20°C for filtration, water in the influent tank was chilled by placing plastic‐wrapped ice packs, which were frozen in the −80°C fridge, in the retentate tank.

### 2.5 Membrane Filtration for Viruses and Surfactants

Each filtration experiments filtered 1 L of virus and surfactant solutions at pH 5 or 7 containing 4.5 × 10^5^ to 5 × 10^8^ TCID_50_/mL of ADV and 4.5 × 10^6^ to 5 × 10^7^ TCID_50_/mL of MHV. During the experiment, water flux was automatically recorded every 10 s using an analytical scale. The flow rate was constantly adjusted to between 1.0 and 1.2 gal per min (GPM). The system pressure was zero psi for all runs through the microfiltration membrane. After each experiment, the solution from the permeate and retentate tank was weighed and the volume was recorded.

Generally, a new membrane was used for each run because the membrane flux was observed to decrease in subsequent runs despite rigorous cleaning. Specifically, when flux was observed to decline, a cleaning process using 2 L of DI water then 3% NaOH with a high flow rate of 1.6–1.8 GPM at 45°C was utilized to attempt to restore the membrane flux. However, this cleaning process was typically not successful in restoring the flux, and as a result, most treatments utilized a new membrane (with the exceptions noted below). Priming water flux for each new experiment was compared to the previous experiments to ensure that the fluxes were similar despite using a new membrane for each experiment.

The treatments used in the study are shown in Table 2. Before filtering viruses, control samples were filtered through the system to observe (a) how flux varied as a function of surfactant presence and pH and (b) virus retention in the absence of surfactants. Control samples without viruses included 1 L of sterile 1X phosphorus buffered saline solution (PBS) (Thermo‐Fisher Scientific, Waltham, Massachusetts, United States) and 1 L of 1 mg/L of SDS or BAC in 1X PBS, with the pH adjusted to 5 or 7 using 1‐M hydrochloric acid and 3‐M sodium hydroxide. Additional control samples containing viruses in the absence of surfactants were also prepared in 1X PBS at pH 5 and 7. The PBS solution was used to preserve virus infectivity. After processing the control samples, multiple runs of viruses and surfactants at various pH were conducted (Table 2). Specifically, sucrose‐concentrated MHV and ADV (2.5 mL of each virus) were added to 1 L of PBS solution, which was then spiked with either 1 mg/L of SDS or BAC. Before each filtration run, 2 mL of water was collected from the influent tank to verify the initial surfactant concentration.

**TABLE 2:** Treatments included in the study.

Treatment	Virus added	Surfactant added	pH	Run #
Control, 1X PBS^a^	None	1 mg/L SDS	5 and 7	1, 4
Control, 1X PBS^a^	None	1 mg/L BAC	5 and 7	2, 3
Control, 1X PBS^a^	None	None	7	5
Control, 1X PBS	ADV and MHV	None	5 and 7	7
1X PBS + virus + SDS^a^	ADV and MHV	1 mg/L SDS	5 and 7	10, 6
1X PBS + virus + BAC	ADV and MHV	1 mg/L BAC	5 and 7	8, 9
Wastewater + SDS	None added	1 mg/L SDS	7	11
Wastewater + BAC	None added	1 mg/L BAC	7	12
Wastewater + virus	ADV and MHV	None added	7	13
Wastewater + SDS + virus	ADV and MHV	1 mg/L SDS	7	14
Wastewater + BAC + virus	ADV and MHV	1 mg/L BAC	7	15

^a^ Experiments were performed with the same membrane.

Immediately after the targeted volume of permeate was collected, typically 500 mL, the pump was stopped. Permeate and retentate volumes were then collected and weighed. A total of 2 mL of permeate and retentate was transferred to HPLC vials for surfactant concentration determination via LC‐MS as described below. The remaining permeate and retentate solution were precipitated using a PEG‐6000‐NaCl concentrator as described below. After completion of a virus and surfactant filtration experiment, the membrane was stored at 4°C for later sectioning and extraction of DNA and RNA for virus quantitation on the membrane. After each filtration experiment, the filtration system was cleaned by pumping 1 L of DI water through the system without a membrane. The tank was brushed and rinsed with DI water to remove any remaining viruses and surfactants.

### 2.6 Virus Purification From Microfiltration Permeate and Retentate

Due to the large volume of permeate and retentate samples collected after microfiltration, the sucrose cushion method, typically used for small‐volume purification, was unsuitable. In this case, a PEG‐6000‐NaCl virus precipitation method was chosen to process and concentrate a large volume of water samples containing viruses. The PEG‐6000‐NaCl concentrator was prepared by dissolving 80 g of PEG‐6000 (Sigma Aldrich) and 14 g of NaCl (Sigma Aldrich) in 80 mL of DI water and 20 mL of 10X PBS solution. The solution pH was adjusted between 7.0 and 7.2, and the final volume was brought to 200 mL using DI water. In this concentrator, the PEG‐6000 was 40% (w/v), and the NaCl was 1.2 M. To precipitate the virus from a large volume of water, one portion of the concentrator was added to three portions of the sample to reach the final PEG‐6000 in solution of 10% and the NaCl final concentration of 0.3 M. Then, the mixture is shaken at 170 RPM for 60 s, followed by constant rocking at 60 RPM for at least 4 h at 4°C to assist virus precipitation. Upon completion, spin the mixture for 60 min at 1600×*g* (3800 rpm) (5910R centrifuge Eppendorf, Hamberg, Germany). After centrifugation, the supernatant was discarded, and the resulting viral pellets were used for DNA extraction and virus culturing. In preliminary experiments, we observed that PEG‐6000 mixed with NaCl did not affect viral infectivity for up to 8 h at 4°C. However, infectivity decreased significantly beyond 8 h or at temperatures above 4°C. Therefore, all samples were incubated with PEG‐NaCl solution for only 4 h at 4°C to ensure viral integrity.

### 2.7 Determination of Surfactants by LC‐MS

The concentration of surfactants SDS and BAC in solution during filtration and in wastewater samples was determined using ultra high‐performance liquid chromatography coupled with triple quadrupole mass spectrometry (i.e., LC‐MS/MS, Agilent 1290/6470). The stationary phase was an Agilent Zorbax Eclipse Plus C18, Rapid Resolution HD (1.8 μm, 50 × 2.1 mm). For both compounds, the flow rate was 0.4 mL/min, and the injection volume was 20 μL.

The mobile phases for SDS were (A) 2‐mM ammonium acetate prepared in 95/5 LC‐MS grade water/acetonitrile and (B) 100% acetonitrile. The gradient method was 70:30 A:B from 0 to 0.20 min, shifted linearly to 40:60 until 1.00 min, shifted to 5:95 until 5 min, shifted to 100% B for 5.5–7.5 min, and shifted back to 70:30 for 8–10 min. The column temperature was maintained at 40°C. SDS was detected via MS in multiple reaction monitoring (MRM) mode with a precursor ion of 265 *m*/*z* and product ions of 97 and 80 *m*/*z*. Other MS parameters included negative ionization mode, collision energies of 20 and 30 eV for 97 and 80 *m*/*z*, cell accelerator voltage of 5 V, nitrogen gas flow of 10 L/min, dwell time of 200 ms, and fragmentor voltage of 70 V.

The mobile phases for BAC were (A) 10 mM of ammonium acetate in LC‐MS water and (B) 100% acetonitrile. The gradient was set at 95:5 A:B (*v*:*v*) from 0 to 2 min, then a slow ramp to 100% B from 2 to 30 min. For the final 3 min, 100% B is held. The injection volume was 20 μL, and the column temperature was 30°C. BAC was detected via MS in MRM with a precursor ion of 304 *m*/*z* and product ions of 58, 91, and 212 *m*/*z*. Other MS parameters include positive ionization mode, collision energies ranging from 20 to 40 eV, and cell accelerator voltage of 5 V. The nitrogen gas flow rate, dwell time, and fragmentation settings were the same as those used for SDS.

Calibration curves for the surfactants were prepared in 1X PBS at pH 7 (*R* ^2^ of 0.9975 for SDS and *R* ^2^ of 0.9986 for BAC), 1X PBS at pH 5 (*R* ^2^ of 0.9850 for SDS and *R* ^2^ of 0.9923 for BAC), and in ultrapure water at pH 7 (*R* ^2^ of 0.9984 for SDS and *R* ^2^ of 0.9938 for BAC). The detection limits for SDS and BAC were 12.5 μg/L.

### 2.8 Scanning Electron Microscopy and Energy‐Dispersive X‐Ray Spectroscopy

Scanning electron microscopy (SEM) images were taken to visualize viruses, surfactants, or other foulants on the microfiltration membrane. Two different SEM technologies were used, with one capable of visualizing biological particles on the membrane (Zeiss‐GEMINI) and one capable of both SEM and energy‐dispersive X‐ray spectroscopy (EDS) scanning (FEI Quanta 600 FE‐SEM) (Hillsboro, Oregon, United States). All membrane samples were prepared by sectioning into 1‐cm^2^ squares and coated with gold/palladium 80%:20% (*w*:*w*) at 3.85‐nm thickness (LEICA ACE 600) (Deerfield, Illinois, United States). Two pieces of membrane for each experiment were prepared, including a center and an edge piece from each membrane. All SEM images used a magnification of 17.5 KX for the general visualization to locate biological materials on the membrane and a magnification of 55.0 KX to visualize and identify biological particles such as viruses. The working distance (Samineni et al. 2019) was 3–6 nm with a 0° tilt angle. The lens was SE2 with an electron high tension (EHT) of 20 kV. Images of clean and used membranes were from different membranes, rather than the same membrane before and after use. For SEM‐EDS, all settings were identical except the working distance changed to 10.6 nm, and the EHT decreased to 10 kV.

### 2.9 Virus Quantification via Quantitative Polymerase Chain Reaction and Culture Assay

Virus abundance was determined using both a culture‐based method and a quantitative polymerase chain reaction (qPCR) method. First, to determine the TCID_50_/mL virus concentrations, an eightfold dilution series was prepared by adding 60–100 μL of each virus to the appropriate cell lines at 90% confluence in duplicate wells (Falcon 96‐well flat bottom microplate, Fisher Scientific, Waltham, MA, United States). After the incubation period, cell apoptosis in each well was assessed by microscopy and then used to determine the TCID_50_/mL. To quantify viral DNA and RNA, qPCR was conducted. Nucleic acids were extracted using a previously published manual DNA and RNA extraction method (Griffiths et al. 2000). For viruses retained on the membranes, the centerpiece of each membrane was cut into 0.5 × 0.5 cm squares, and 10 pieces were used for nucleic acid extraction.

For the qPCR, all oligonucleotides used as positive controls, primers, and probes were purchased from Integrated DNA Technologies (IDT‐DNA, Coralville, Iowa, United States). RT‐qPCR and qPCR assays were conducted using Quant Studio 3 (Thermo‐Fisher Scientific, Waltham, Massachusetts, United States). All qPCR and RT‐qPCR runs included the corresponding positive control (oligonucleotides) and negative control (molecular grade water). For the RT‐qPCR assay, the MHV positive control was 5′GGAACTTCTCGTTGGGCATTATACTTTTTTACATGCTACGGCTCGTGTAACCGAACTGTTTTTTTATGTTGTGAAAATGATAATCTTGTGGT‐3′. The MHV RT‐qPCR analysis was performed in duplicate in 25‐μL reaction mixtures, which contained 20 μL of master mix (TaqPath 1 step RT‐qPCR MM, CG, Thermo‐Fisher Scientific, Waltham, Massachusetts, United States) and 5‐μL MHV RNA samples. The MHV primer and probe sequences were used as previously reported (Gill et al. 2022). The thermocycling conditions for MHV RT‐qPCR were 50°C for 15 min, 95°C for 2 min, and 40 cycles of 95°C for 10 s followed by 60°C for 30 s. A sixfold dilution series of the positive control oligonucleotide was used to generate an MHV standard curve with a reaction efficiency of 89.1% and an *R* ^2^ of 0.9936. The ADV positive control was 5′CACTCATATTTCTTACATGCCCACTATTTTTTTAGGAAGGTAACTCACGAGAACTAATGGGCCATTTTTCAATCTATGCCCAACAGGCC‐3′. The ADV qPCR analysis was performed in duplicate 25‐μL reaction mixtures, which contain 20 μL of master mix (TaqMan Fast Advanced Master Mix, Thermo‐Fisher Scientific, Waltham, Massachusetts, United States) and 5 μL of ADV DNA. The ADV primer and probe sequences were previously reported (Ebner et al. 2006). The thermocycler conditions were 95°C for 5 min, and 40 cycles of 95°C for 15 s followed by 60°C for 30 s. A sixfold dilution series of the positive control oligonucleotide was used to generate an ADV standard curve with a reaction efficiency of 90.4% and an *R* ^2^ of 0.9998.

### 2.10 Statistical Methods

A mass balance approach was used to evaluate virus abundance in the initial solution, permeate, and retentate solutions and retain on the membrane, based on both culture and qPCR results. The mass balance for viable viruses was calculated using Equation (1), whereas qPCR virus counts were calculated using Equation (2).

(1) $${C_{I}}^{*}\;V_{I}={C_{P}}^{*}V_{P}+{C_{R}}^{*}\;V_{R}$$

(2) $${C_{I}}^{*}\;V_{I}={C_{P}}^{*}V_{P}+{C_{R}}^{*}\;V_{R}+{C_{M}}^{*}\;A_{M}$$

In Equations (1) and (2), *C*_*I*_ represents the initial virus concentration, determined either by TCID_50_/ml or by qPCR in log gene copies/mL. The terms *C*_*P*_, *C*_*R*_, and *C*_*M*_ denote virus abundance in the permeate (*C*_*P*_), the retentate (*C*_*R*_), and retained per cm^2^ of the membrane (*C*_*M*_). The volume terms *V* refer to the initial volume (*V*_*I*_), total permeate volume (*V*_*P*_), or the total retentate volume (*V*_*R*_). Finally, *A*_*M*_ refers to the area of the membrane available for filtration in cm^2^.

To assess the effectiveness of membrane filtration, virus log reduction (VLR) was calculated as the difference between the initial virus gene copy mass and that of the permeate, as shown in Equation (3).

(3) $$\mathrm{VLR}=\mathrm{Log}\;\left(C_{I}\right)-\mathrm{Log}\;\left(C_{P}\right)$$

For estimating the membrane rejection percentage for qPCR analysis, the concentration of nucleic acids retained on the membrane was included as shown in Equation (4).

(4) $$\text{Membrane rejection}={\left(1-C_{P}/\left(C_{R}+C_{M}\right)\right)}^{*}\;100$$

## 3 Results

### 3.1 Virus Harvesting and Purification

Several studies were conducted to verify virus infectivity after various holding times, conditions, and purification methods. First, the impact of freeze–thaw cycles on virus infectivity in the virus stock solution was tested. Each freeze and thaw cycle of the virus stocking solution was found to decrease the ADV virus count between 10^2^ and 10^3^ TCID _50_/mL. MHV was more tolerant of freeze and thaw cycles and decreased in virus count by 10^1^ TCID_50_/mL. Second, virus infectivity was assessed during extended incubation with cell lines. MHV infectivity significantly decreased beyond 24 h, with no detectable infectious viruses remaining after 48 h. Conversely, ADV remained infective in cell culture for up to 48 h. The third test evaluated virus titer increase after sucrose cushion purification. This method increased the MHV viral titer from 10^2^ to 10^5^ TCID _50_/mL and the ADV viral titer from 10^7^ to 10^10^ TCID _50_/mL. The fourth test examined long‐term storage at −80°C. Both MHV and ADV maintained viability at 10^5^ TCID50/mL after storage for over a month without freeze–thaw cycles, demonstrating stable infectivity in cell lines. Finally, the PEG‐6000‐NaCl precipitation method was tested for efficacy. With this method, MHV titer increased from 10^2^ TCID_50_/mL to 10^5^ TCID_50_/mL, and ADV increased from 10^7^ TCID_50_/mL to 10^9^ TCID_50_/mL for filtration retentate. Both viruses remained stable in PEG‐6000‐NaCl for up to 8 h at 4°C. Over 90% of ADV input viruses were recovered following PEG‐6000‐NaCl precipitation, though MHV recovery showed variability across tests. Residual PEG‐6000‐NaCl did not induce cell necrosis or interfere with virus infectivity in cell cultures.

### 3.2 Water Flux and Surfactant Mass Balance

Water flux through the microfiltration membrane was influenced by the presence of surfactants. When surfactants or PBS solution was sequentially filtered through the same membrane, the flux decreased from 2 to 3 g/s initially to a steady state flux between 0.4 and 1.5 g/s in subsequent runs (Figure S1). Rigorous cleanings between runs with DI water and NaOH were not able to recover membrane flux, likely due to the irreversible sorption of surfactants to the membrane surface. Sorption of the surfactants to the membrane, causing fouling of the pores, was observed by SEM when very high surfactant concentrations were used (e.g., 50 mg/L BAC or 100 mg/L SDS, Figures S2–S4). Further sorption of the surfactants to the membrane was inferred by observing differences between initial surfactant concentrations and concentrations in the permeate and retentate when using lower 1 mg/L surfactant concentrations. Initial surfactant concentrations in the virus filtration studies were 0.86 ± 0.73 mg/L for SDS and 0.58 ± 0.50 mg/L for BAC. Generally, less than 1% of the SDS was measurable in the retentate and permeate after filtration with viruses. SDS was assumed to be sorbed to the membrane and the stainless steel parts of the membrane filtration apparatus. Less BAC was lost to the system compared to SDS. As a percentage of the initial BAC added, the permeate averaged 28.9% ± 40% and the retentate averaged 29.3% ± 22% among all the treatments with BAC. The BAC loss to the membrane and filtration system surfaces averaged 41.8%. Given the reduction in flux in subsequent runs with the same membrane, a new membrane was used for each additional virus rejection study (Table 2). When using a new membrane for each study, a stable water flux of 3 g/s was achieved (Figure S1).

### 3.3 Virus Microfiltration Rejection Influenced by Surfactants

**FIGURE 2:**
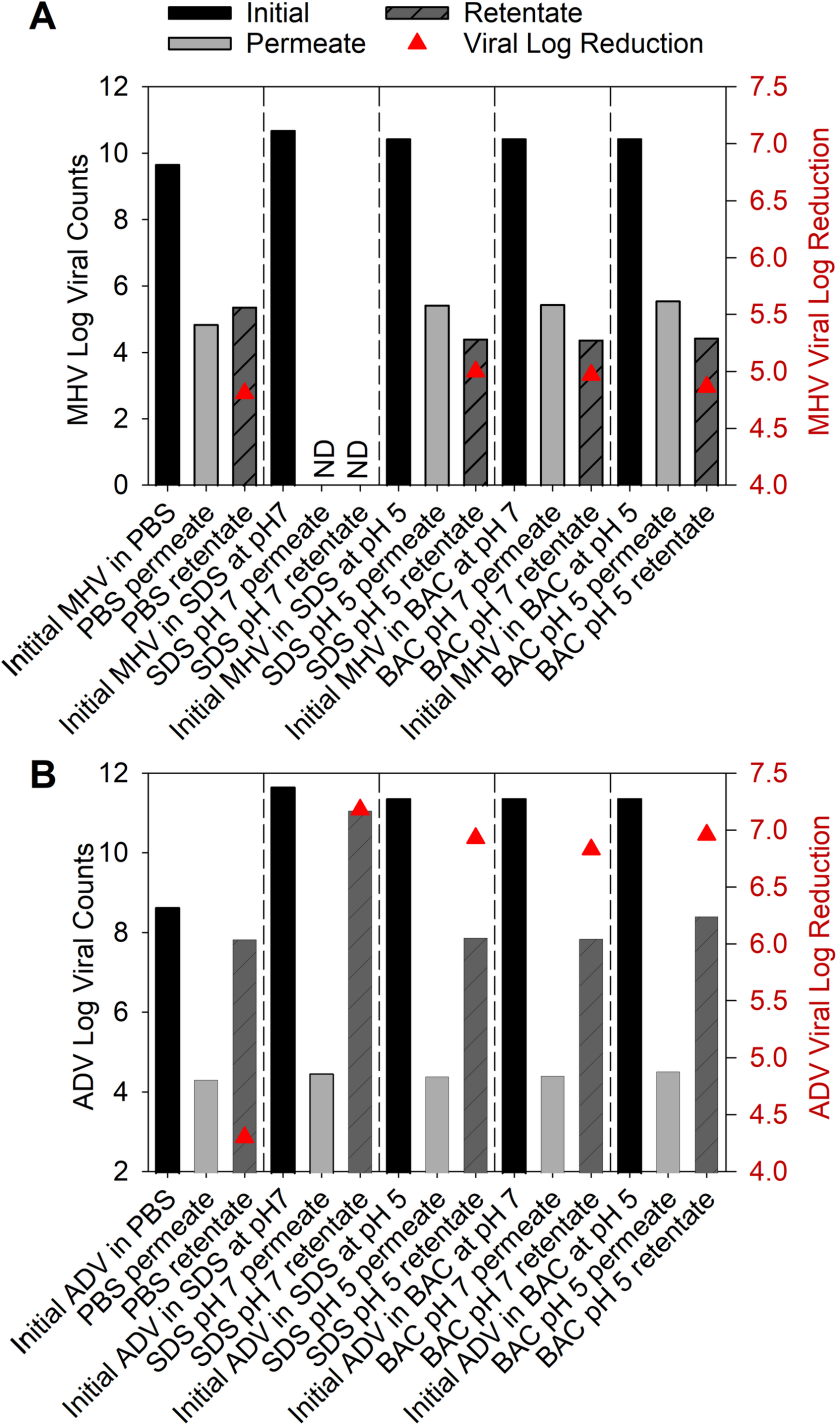
Culturable virus rejection (triangles) and viral counts (bars) for MHV (A) and ADV (B) in PBS with and without surfactants. Culturable MHV was not detected in the permeate nor retentate of the SDS at pH 7 study and is indicated with “ND.”

Culturable virus rejection with and without the presence of surfactants is shown in Figure 2. The black bar represents the initial virus concentration, purified using sucrose. The gray and striped gray bars represent the viruses recovered from the permeate and retentate tanks after filtration using PEG‐6000‐NaCl. The red triangle represents the viral log reduction calculated with Equation (3). In the absence of surfactants, the microfiltration membrane with an average pore size of 200 nm rejected 4.3 log ADV and 4.8 log MHV, which have diameters of 80–120 nm for MHV and 90 nm for ADV. When surfactants were added, the rejection of ADV increased significantly to between 6.8 and 7.1 log. The rejection of MHV also increased when surfactants were present to between 4.9 and 5.0 log. The increased rejection of ADV and MHV did not appear to be related to pH, as only for BAC at pH 5 compared to BAC at pH 7 did the MHV viral log reduction increase by 0.1 log. No infective MHV viruses were detected in the permeate or retentate for SDS at pH 7, likely due to the low water flux observed in Figure S1.

There was a significant loss of infectivity of the enveloped virus MHV during filtration as only 0.001%–0.006% of the MHV was culturable in both the permeate and retentate. In contrast, 16% of the nonenveloped ADV was still infective in permeate when no surfactants were present. However, the infectivity of the nonenveloped ADV was significantly lower when the ionic surfactants were present, dropping to 0.03%–0.1% in the retentate. Less than 0.004% of the ADV from the original virus titer was found in the permeate and was infective.

**FIGURE 3:**
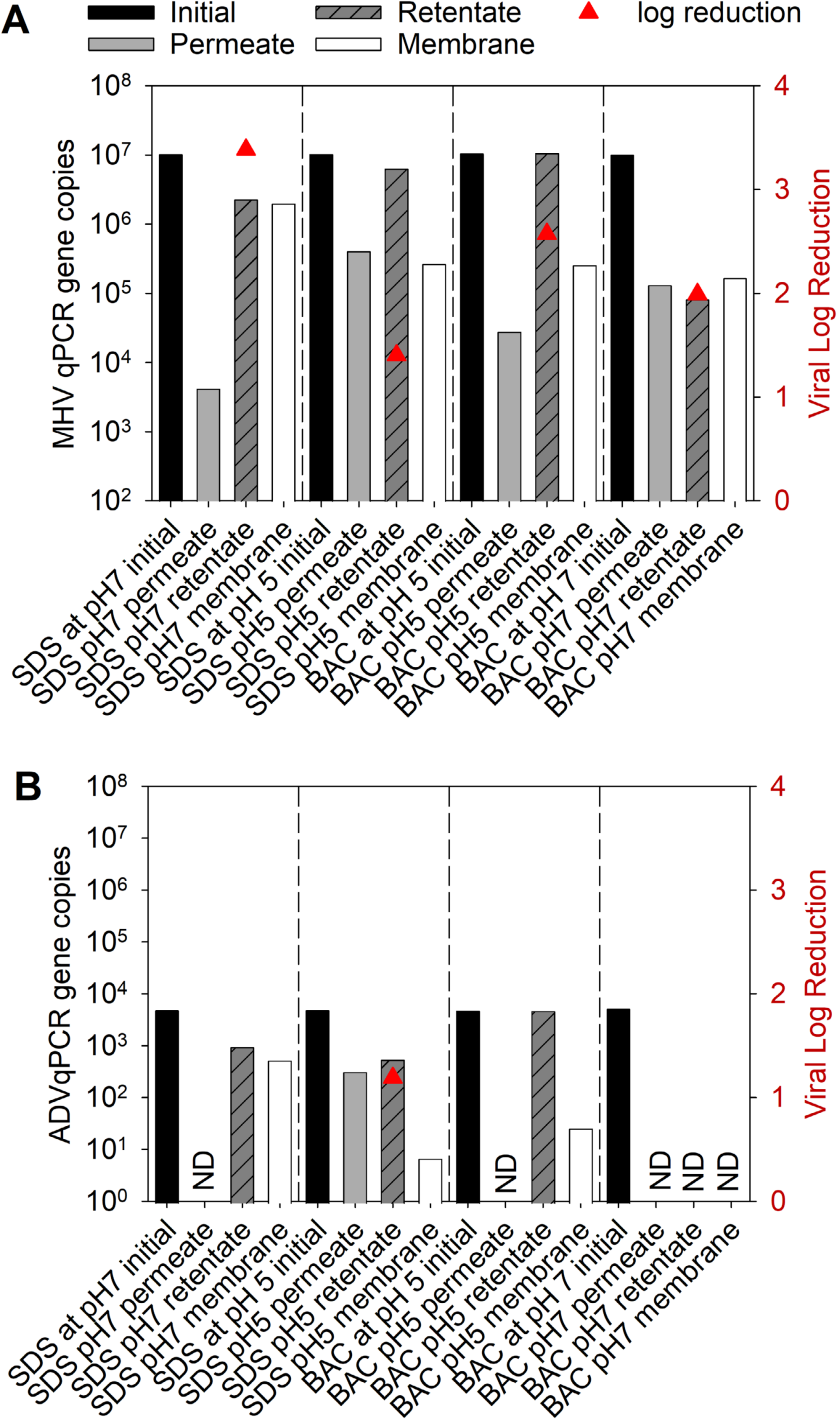
Virus nucleic acid rejection (triangles) and viral counts (bars) for MHV (A) and ADV (B) in PBS with surfactants. ADV was not detectable in the permeate in BAC studies nor in the SDS pH 7 study and is indicated with an “ND.”

To assess the rejection of infectious and noninfectious viruses, qPCR‐based quantification of viral gene copies in the initial solution, permeate, and retentate was conducted. Further, the abundance of virus nucleic acids retained on the membrane was determined. The rejection of MHV determined by qPCR is shown in Figure 3A. The log reduction in MHV nucleic acids during microfiltration was lower than that found by culture‐based methods, ranging from 1.4 to 3.4 log reduction by qPCR compared to 4.9 to 5 LRV for infective viruses. Generally, good recovery of the MHV nucleic acids was observed during microfiltration based on mass balance calculations. MHV nucleic acids in the retentate, permeate, and membrane as compared to the initial gene copies added ranged from a low of 4% for the BAC at pH 7 study to a high of 104% for the BAC at pH 5. The MHV nucleic acid recovery for the SDS studies was 41% and 68% for pH 7 and 5, respectively. In contrast, ADV nucleic acids were less likely to be detected in the permeate than MHV (Figure 3B). Recovery of ADV gene copies, as calculated from mass balances of gene copies in the permeate, retentate, or recovered from the membrane, ranged from 99% for BAC at pH 5 treatments to 18%–50% for SDS at pH 5 and 7 treatments, respectively. Incomplete or low recovery of the ADV gene copies in qPCR analysis could be due to (1) loss of DNA during extractions, (2) qPCR reaction inefficiencies due to the presence of inhibitors, or (3) degradation of free nucleic acids by surfactants. No recovery control was included in the nucleic acid extraction, which could have alleviated any concerns regarding inhibitory compounds and loss of DNA during extraction. Overall, a significant retention of the virus or free virus nucleic acids on the membrane, regardless of treatment, was observed.

### 3.4 Virus Microfiltration Rejection in Wastewater Containing Surfactants

Studies were undertaken to determine virus rejection by the microfilter when surfactants and viruses were added to wastewater effluent. No suitable method was available for culturing ADV and MHV from wastewater; therefore, only qPCR results are presented herein. Further, no effort was made to adjust the pH of the real wastewater effluent solution, and the pH was approximately 7 for all experimental conditions (see Table 2). Initial virus concentrations in the wastewater effluent used were low, approximately 4 gene copies/mL for ADV and 311 gene copies/mL for MHV. Before spiking in surfactants to the wastewater effluent, the surfactant concentrations were estimated to be 4.7 ug/L for SDS and 24.9 ug/L for BAC.

**FIGURE 4:**
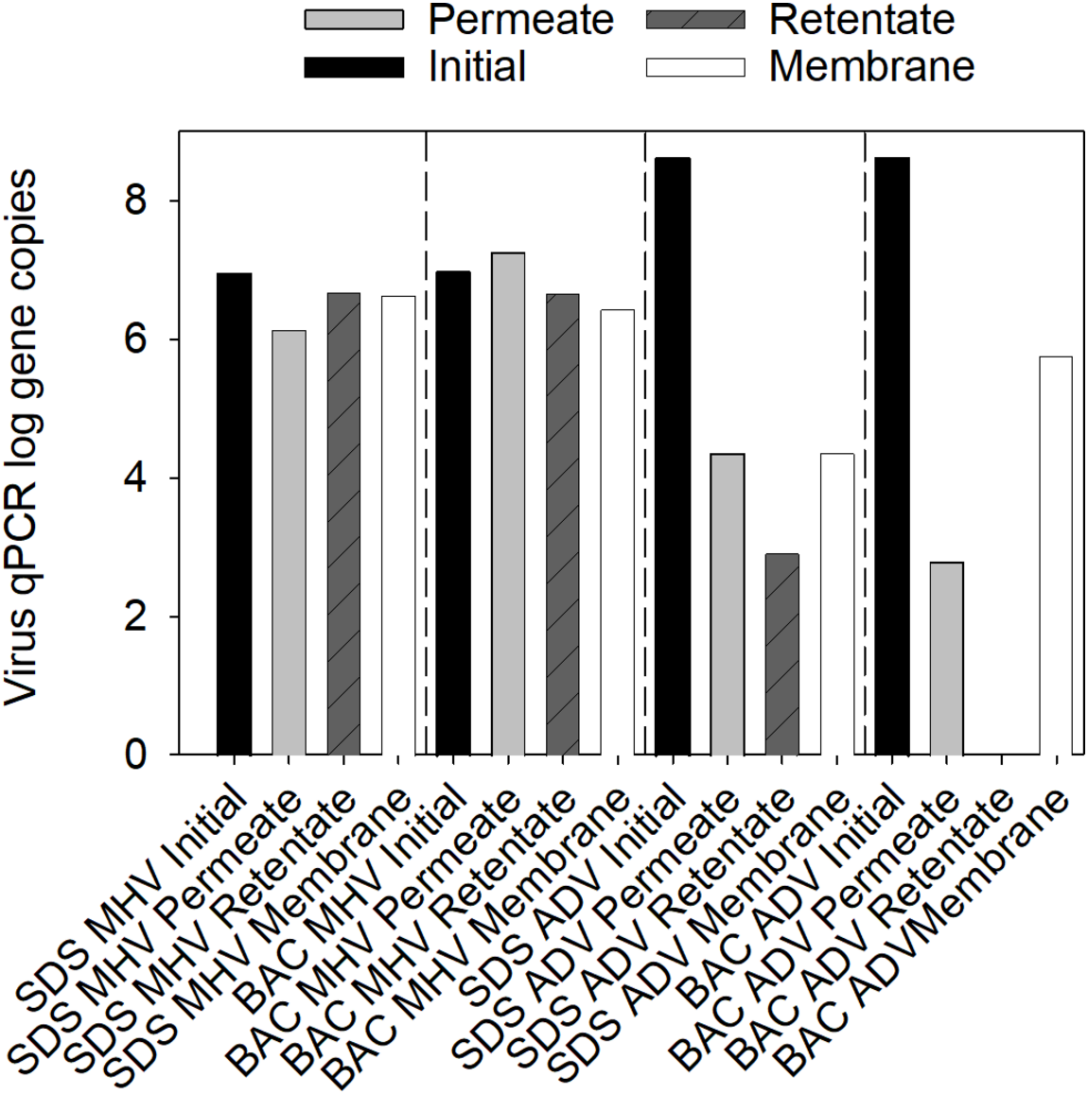
Virus mass balance in the permeate, retentate, and retained on the membrane determined by qPCR. Viruses and surfactants were spiked into wastewater effluent prior to filtration.

When using real wastewater effluent, less MHV rejection was observed (Figure 4) compared to studies with virus in 1X PBS and ranged from 0.8‐log rejection for SDS‐amended experiments to no rejection for BAC‐amended experiments. ADV rejection in real wastewater via microfiltration varied from 4.3 to 5.8 log for experiments containing SDS and BAC, respectively.

### 3.5 SEM and EDS

**FIGURE 5:**
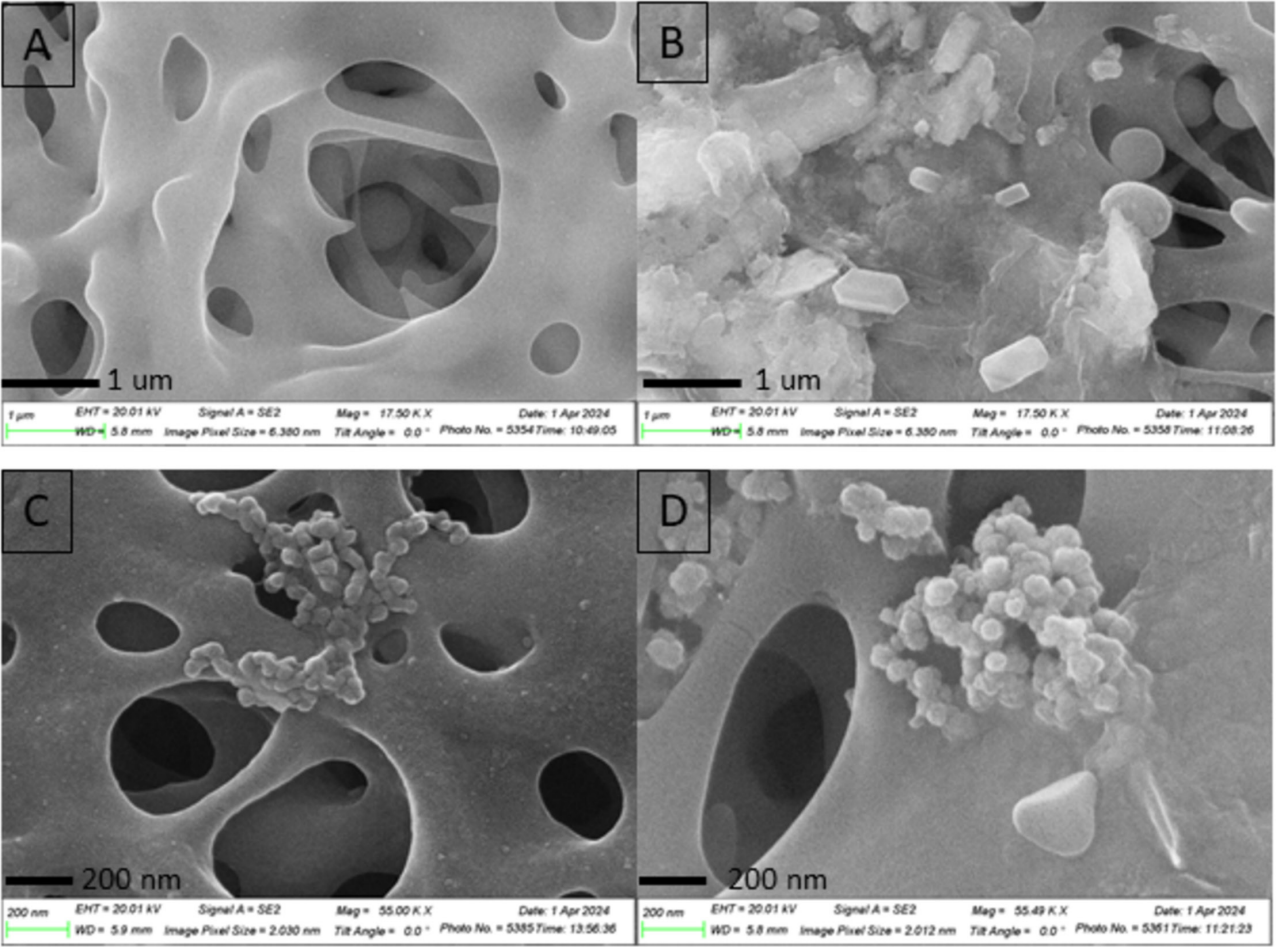
SEM images analysis of PES 020 microfiltration membrane before and after filtration. (A) clean membrane; (B) 1X PBS filtered membrane; (C) SDS 1 mg/L and viruses in 1X PBS solution at pH 5 with viruses center; and (D) BAC 1 mg/L and viruses in 1X PBS solution. A and B have magnification at 17.50 kX and 1.0 um scale bar. C and D have 55.0 kX magnification and 200‐nm scale bars.

The extent of surfactant and virus fouling on the microfiltration membrane was visualized by SEM (Figure 5). Both “new” and “used” membranes were imaged. Additional SEM images are provided in the Supporting Information. The new membrane SEM image showed the porous nature of the PES 020 membrane created by the phase inversion construction technique. The membrane pores were in different shapes and sizes, some of which were significantly larger than the nominal cutoff size of 0.2 um (Figure 5A). Because all surfactants and viruses were prepared using 1X PBS solutions, SEM images were obtained after filtering PBS to visualize any fouling from salt precipitates. The PBS salt precipitates exhibited clear edges and rectangular shapes with particle sizes up to 500 nm (Figure 5B). Previous membrane filtration experiments using a higher concentration of surfactants (SDS 100 mg/L or BAC 50 mg/L) exhibited significant membrane fouling as shown by SEM imaging (Figures S2–S4). These surfactants could not be removed using either DI water or NaOH cleaning methods, as the flux did not recover postcleaning, suggesting the fouling was irreversible. After each filtration experiment, the microfiltration membrane was imaged using SEM and SEM‐EDS so that the viruses and any materials fouling the membrane could be easily visualized and identified. Clusters of viruses were visible in both Figure 5C and Figure 5D in the expected size ranges. Precipitation of surfactants or phosphates from the PBS is also visible in Figure 5D.

**FIGURE 6:**
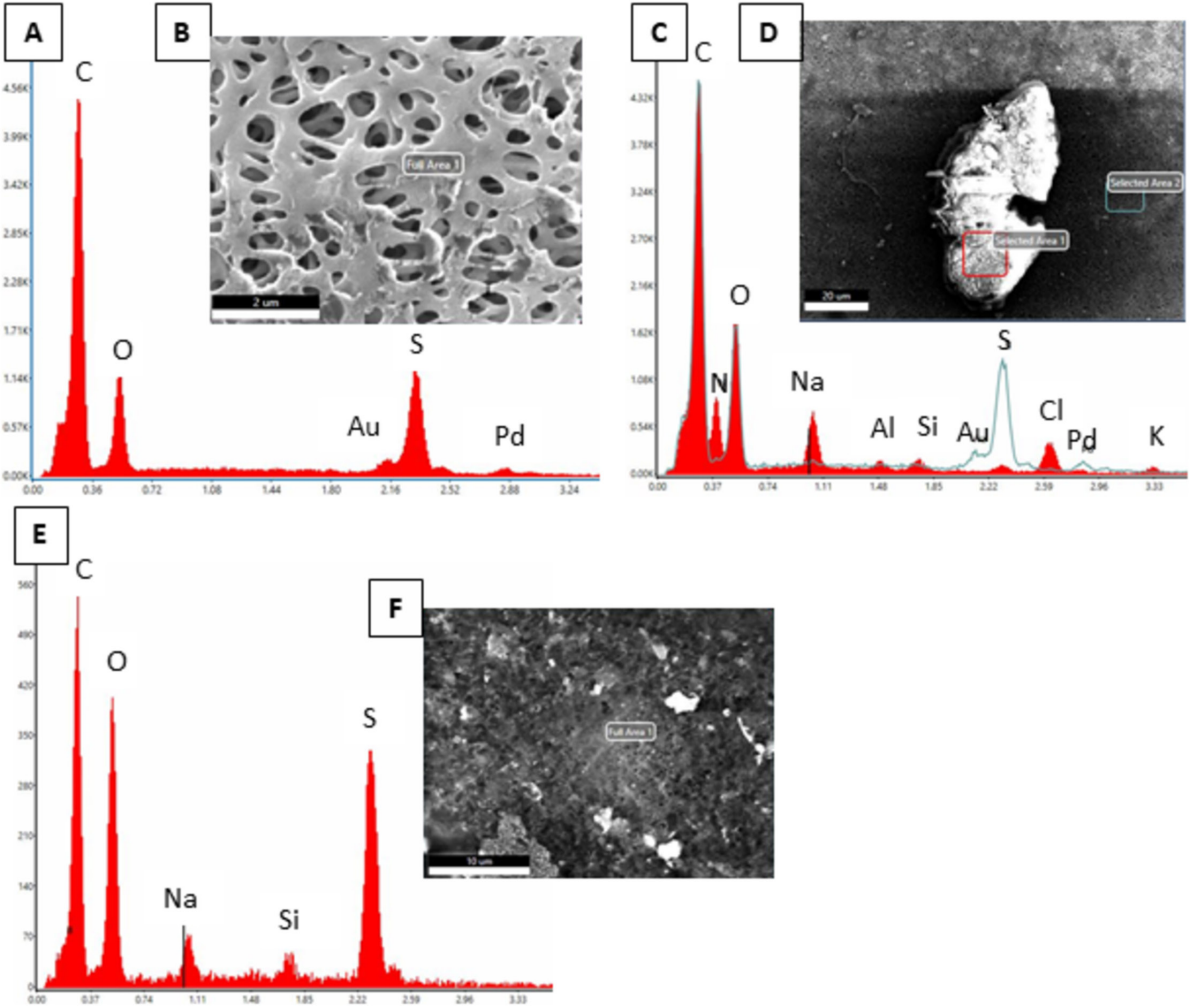
SEM‐EDS analysis of the PES 020 membrane. (A) EDS report of the clean membrane and (B) Quanta SEM image. Scale bar = 2 um. (C) EDS report and (D) SEM image for the virus filtered with 1 mg/L BAC at pH 7. The red square was Area 1, which indicated organic fouling. Green Area 2 was the control area with no particles visible. Scale bar = 20 um. (E) EDS report and (F) SEM image of 50 mg/L BAC filtered fouled membrane. Scale bar = 10 um.

To verify if viruses were present on the membrane and not just surfactants or PBS precipitates, SEM‐EDS was conducted (Figure 6). The clean PES 020 membrane primarily had the elements C, O, and S (Figure 6A,B), whereas the BAC fouled membrane also contained Na and Si in addition to C, O, and S (Figure 6E,F). Although BAC also contained nitrogen in the functional head, no N was detectable in the EDS above the background level. When the viruses were also present (Figure 6C,D), N was clearly detected in the EDS likely associated with the nitrogen in the envelope or capsid. Additional elements detected when BAC and the virus were filtered include Cl and Na. The likely source of the Na was from the 1X PBS solution that contained sodium. The Au/Pt coating was also detected on the membrane, which was expected as the membrane was coated using a thin layer of Au/Pt.

## 4 Discussion

The culture assay and TCID_50_/mL calculator were used herein to quantify the number of viable viruses in both permeate and retentate as they are considered the “gold standard” to determine the virus concentration (Arango et al. 2013). Because viable viruses in treated effluent and receiving waters can affect water quality and directly affect human health, culture‐based results must be accurate and sensitive (Arango et al. 2013; Gall et al. 2015). However, culture‐based techniques are very expensive to maintain and require an aseptic environment for all procedures to prevent contamination by other organisms (Arango et al. 2013). Additionally, the long incubation period required for viruses to infect cell lines (MHV 24 h and ADV 48 h), in addition to the time to grow cell lines (3 to 5 days), makes this method less practical for some research applications. For this study, because the wastewater secondary effluent contained additional materials other than viruses, it was not feasible to do a cell culture study targeting just the spiked viruses in both permeate and retentate samples.

In addition to traditional cell culture, PCR‐based techniques are widely used to quantify microorganisms in a highly sensitive, specific, and reliable manner (Khan et al. 2020). For this study, it was important to know the total amount of viruses in permeate, retentate, and membrane regardless of the virus viability. In this case, RT‐qPCR and qPCR were used to quantify the total amount of viruses before and after microfiltration. Even though qPCR‐based techniques were able to handle a large number of samples simultaneously, the sample preparation process, particularly for virus precipitation and virus DNA/RNA extraction, is time consuming (Khan et al. 2020).

Previous literature indicated that viruses should be stable in PEG‐8000 solution and that up to 90% of input infectious viruses could be recovered after PEG purification (Lo and Yee 2007). Additionally, the residual PEG‐8000 in the purified viruses did not induce toxicity in the cell line. Because we used PEG‐6000 instead of PEG‐8000 for all filtration experiments, our results indicated that (1) diluted PEG‐6000 solutions did not cause cell toxicity for both cell lines used and (2) only 50% or fewer of the recovered viruses remained viable when tested via cell culture (Figure 3) (Polson et al. 1972). Although some studies suggested storing viruses in PEG‐8000 solution overnight before extraction could enhance recovery (Lo and Yee 2007), our results showed that the storage time should be less than 8 h to avoid virus inactivation. The storage time and process should be tailored to the specific viruses being studied.

The microfiltration membrane results showed that it was efficient to use a microfiltration membrane to remove both viable MHV and ADV viruses. Literature indicated that the efficiency could be attributed to mechanisms such as size exclusion, forming fouling layers, or electrostatic interactions described by the DLVO theory (Sinclair et al. 2018; Agmo Hernández 2023; Yasui et al. 2024). In addition to electrostatic interactions, the MF also achieved virus removal due to adsorption and hydrophobic interaction with the membrane surface (Sinclair et al. 2018). Previous microfiltration studies using nylon and PTFE membranes on the bacteriophage MS2, Qβ, phiX174, and phi6 have confirmed that a hydrophilic membrane achieved the highest log reduction value at the pH when the membrane carried a slightly positive charge based on solution pH (Yasui et al. 2024). In our case, because the PES membrane was negatively charged, the BAC in the solution could introduce positive charges to the solutions and neutralize some or all of the charges on the membrane surface, leading to a higher log reduction value even with both viruses containing a negative surface charge at either pH 5 or 7 (Mahdi et al. 2019). The same concept had been confirmed using polyethyleneimine (PEI), which had a cationic polymer coat on the top of a PES microfiltration membrane. By adding positive charges, prior reports suggested microfiltration can increase MS2 reduction by ≥ 3 log_10_ units (≥ 99.9%) with around 22% loss of permeability (Sinclair et al. 2018). Additionally, research on using MF and slow sand filters treated pepper mild mottle virus (PMMoV) in drinking water and virus quantification using qPCR indicated that the hydrophobic PVDF (polytetrafluoroethylene) MF could remove viruses from 0.0 to 0.9 log10 with around 40% of viruses below the qPCR detection limit (Canh et al. 2019). Because most of the enteric viruses, including ADV, had negative surface charges with their IEP less than 7, it was previously concluded that MF was not an effective way to remove viruses in drinking water (Canh et al. 2019). However, our results indicated that in most permeate, ADV could not be detected using qPCR (Figure 3B). This was typically because the ADV concentration was likely below the qPCR detection limit, which might be a false negative (Canh et al. 2019). Alternatively, ADV might not have been detected by qPCR due to the low initial virus concentration in the solution (Canh et al. 2019).

Limitations to this study included a lack of replication of the membrane filtration experiments due to the complexity and duration of each experiment. From virus harvest to purification, microfiltration, extraction, and qPCR, each condition was tested once. However, for consistency, all sample materials were pooled and aliquoted to ensure uniformity. For instance, purified virus stocks were thoroughly mixed and then distributed in equal aliquots. The same procedure was used for preparing surfactant and PEG solutions.

Previous literature had demonstrated that SDS had an effect on the solubilization of the plasma membrane by targeting its phospholipid content (Tukmachev et al. 1979). This is supported by other studies in our laboratory that showed that SDS preferentially interacted with the MHV spike protein and envelop lipids (data not shown). SDS was also used as a gelatin media outside of the PES membrane to enhance its hydrophilicity (Alsari et al. 2001). Given that the PES membrane itself had a negative surface charge, the addition of SDS to the solution, along with viruses, increased the negative charges in the system. In an aqueous solution, increasing the solution ionic strength, the SDS decreased the solubilization effects on the membrane surface due to the increase in the SDS micelle volume (Tukmachev et al. 1979). Because all filtration experiments used PBS buffer, it was assumed that more SDS was absorbed on the PES membrane surface. Sorption of SDS decreased the size of the membrane pores, thereby promoting virus rejection via size exclusion, as shown in Figures 2B and 3 (Yasui et al. 2024).

Scanning electron microscopy (SEM) was used to visualize the viruses and surfactants adsorbed on the microfiltration membrane surface. Literature showed that although SEM was rarely used for microbial detection, especially to visualize viruses, it is useful only in two cases: (1) when biological samples are not culturable and (2) in filtration studies to identify pathogens on the surface (Khan et al. 2020). Herein, it was useful to visualize the viruses on the membrane surface because the viruses on the membrane were not culturable. Previous studies used SEM to visualize the membrane, which coated the membrane with 27‐ and 18‐nm thickness metal, and indicated that the thickness of the metal coat did not affect the pore size (Golding et al. 2016). In this research, we observed that the thickness of the metal coat on the membrane affected the pore size. Because the materials adsorbed on the membrane surface were not sufficiently conductive to give a clear contrast and resolution, a thin layer of gold/platinum 3.8‐nm thickness was coated on the membrane surface (Khan et al. 2020). This specific metal coating was chosen to differentiate it from the PES membrane, which consists of carbon (C), hydrogen (H), oxygen (O), and sulfur (S) (Giesa and Schmidt 2001). Given that most of the SEM instruments could scan up to a depth of 1 um from the surface, it was assumed that 3.8 nm was thin enough not to affect the image quality (Giurlani et al. 2020).

## 5 Conclusion

This study evaluated the effectiveness of a microfiltration batch system in removing viruses in the presence of ionic surfactants. Both viable and nonviable virus concentrations were determined using cell culture and qPCR under varying experimental conditions. In the absence of surfactants, the MF system rejected viable viruses on the order of 4.3 log ADV and 4.8 log of MHV. The MHV and ADV viable virus rejection increased between 4.9–5.0 and 6.8–7.1, respectively, when surfactants were added. Both infective MHV and ADV in permeate were significantly low, with 0.001%–0.006% of the MHV and 16% of the ADV remaining infective after filtration without surfactants. The infectivity of ADV dropped to 0.03%–0.1% in retentate and 0.04% in permeate when surfactants were present. Visualization of the membrane surface using SEM‐EDS showed that virus particles were present on the membrane. When BAC was also filtered, viruses were more frequently observed on the membrane, likely due to the neutralization of the negative virus surface and/or membrane filter surface charges by the positively charged BAC. Finally, surfactants contributed to fouling the membrane surface, which reduced the membrane pore size and increased virus membrane rejection. The results of this study help inform understanding of log reduction values for enveloped and nonenveloped viruses during membrane filtration in the presence of surfactants.

## Author Contributions

**Makayla Loey:** methodology, investigation, data curation, formal analysis, visualization, writing – original draft. **Damien Redder:** investigation, data curation. **Emily L. Marron:** conceptualization, funding acquisition, writing – review and editing. **Jennifer Weidhaas:** conceptualization, funding acquisition, supervision, project administration, writing – review and editing.

## Conflicts of Interest

The authors declare no conflicts of interest.

## Supporting information


**Figure S1:** (A) Decreasing flux observed in sequential filtration experiments using the same membrane with DI water and NaOH cleaning between each run. (B) Steady water flux observed when a new membrane was used for each run.
**Figure S2:** SEM‐EDS analysis of the membrane used in this study.
**Figure S3:** SEM‐EDS analysis showing fouling of membrane during filtration of 100 mg/L SDS.
**Figure S4:** SEM‐EDS analysis showing fouling of membrane during filtration of 50 mg/L BAC.

## Data Availability

The data that support the findings of this study are available from the corresponding author upon reasonable request.
