# Supplementary material for: Influence of Ionic Surfactants on Microfiltration of Enveloped and Nonenveloped Viruses
Source: Water Environ Res. 2025 Sep 2;97(9):e70171. doi: 10.1002/wer.70171 (PMC12402861; doi:10.1002/wer.70171)
Supplement: Supplementary file 1 — Figure S1: (A) Decreasing flux observed in sequential filtration experiments using the same membrane with DI water and NaOH cleaning between each run. (B) Steady water flux observed when a new membrane was used for each run. Figure S2: SEM‐EDS analysis of the membrane used in this study. Figure S3: SEM‐EDS analysis showing fouling of membrane during filtration of 100 mg/L SDS. Figure S4: SEM‐EDS analysis showing fouling of membrane during filtration of 50 mg/L BAC. [file WER-97-e70171-s001.docx]

**Supplemental information**

for

**Influence of ionic surfactants on microfiltration of enveloped and non-enveloped viruses**

Makayla Loey^1^, Damien Redder^2^, Emily Marron^3^, Jennifer Weidhaas ^4^ *

^1^ University of Utah, Department of Civil and Environmental Engineering, 110 Central Campus Drive, Suite 2000, Salt Lake City, UT 84112, [makayla.loey@utah.edu](mailto:makayla.loey@utah.edu)

^2^ University of Utah, Department of Civil and Environmental Engineering, 110 Central Campus Drive, Suite 2000, Salt Lake City, UT 84112, [u1246946@utah.edu](mailto:u1246946@utah.edu)

^3^ University of Hawaii, Water Resources Research Center, 2540 Dole Street,

Honolulu, HI 96822, [marron@hawaii.edu](mailto:marron@hawaii.edu)

^1^ University of Utah, Department of Civil and Environmental Engineering, 110 Central Campus Drive, Suite 2000, Salt Lake City, UT 84112, [jennifer.weidhaas@utah.edu](mailto:jennifer.weidhaas@utah.edu), 801-585-1228

* Corresponding author: Jennifer Weidhaas

**Table of contents**

Supplemental Figure 1. (A) Decreasing flux observed in sequential filtration experiments using the same membrane with DI water and NaOH cleaning between each run. (B) Steady water flux observed when a new membrane was used for each run.

Supplemental Figure 2. SEM-EDS analysis of the membrane used in this study.

Supplemental Figure 3. SEM-EDS analysis showing fouling of membrane during filtration of 100 mg/L SDS

Supplemental Figure 4. SEM-EDS analysis showing fouling of membrane during filtration of 50 mg/L BAC

~~~~

Supplemental Figure 1. (A) Decreasing flux observed in sequential filtration experiments using the same membrane with DI water and NaOH cleaning between each run. (B) Steady water flux observed when a new membrane was used for each run.


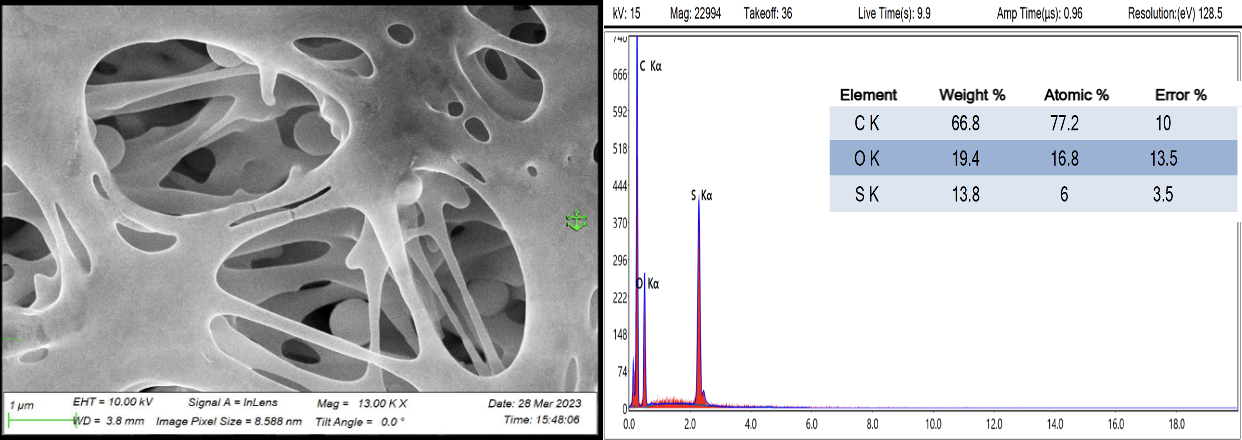


Supplemental Figure 2. SEM-EDS analysis of the membrane used in this study.


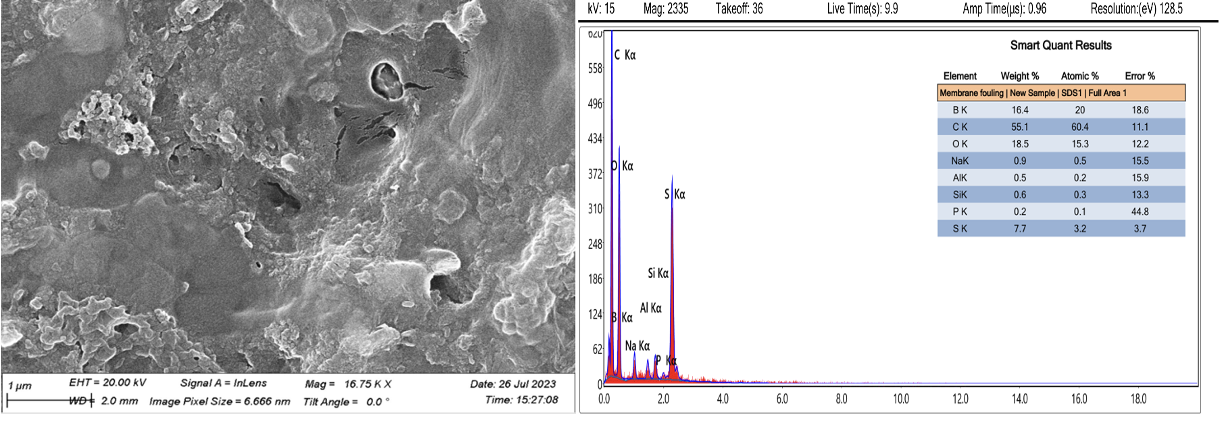


Supplemental Figure 3. SEM-EDS analysis showing fouling of membrane during filtration of 100 mg/L SDS.


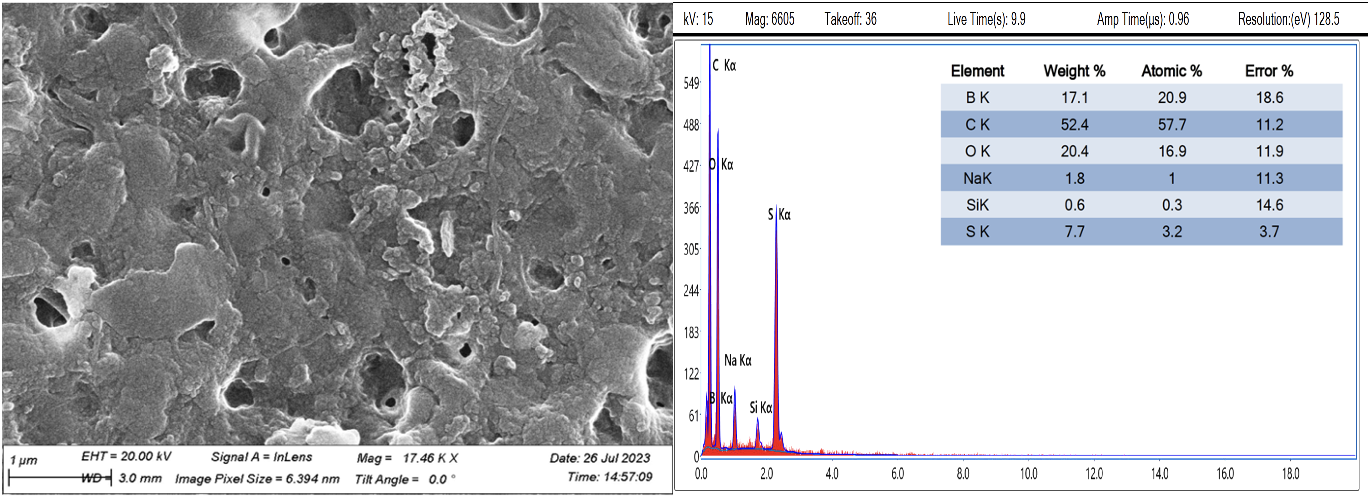


Supplemental Figure 4. SEM-EDS analysis showing fouling of membrane during filtration of 50 mg/L BAC.
